# The Association of IL‐17RC Polymorphisms rs708567 and rs76999397 With Acute Lymphoblastic Leukaemia

**DOI:** 10.1111/iji.70012

**Published:** 2025-08-31

**Authors:** Ali Aljuaimlani, Lamjed Mansour, Jameel Al‐Tamimi, Jamilah Alshammari, Safa A. Alqarzae, Fadwa M. Alkhulaifi, Suliman Alomar

**Affiliations:** ^1^ Department of Zoology College of Science King Saud University Riyadh Saudi Arabia

**Keywords:** genetic | interleukin receptor C | polymorphism | T lymphocytes

## Abstract

Background: Acute lymphoblastic leukaemia (ALL) is characterized by the clonal proliferation of immature lymphoid precursors in the bone marrow or peripheral blood. This study investigates whether genetic polymorphisms in *IL‐17RC* are associated with an increased risk of ALL in the Saudi population.

Methods: This case‐control study included 95 patients with ALL and 95 matched controls. Genetic polymorphisms and their associations with ALL risk were identified using logistic regression analysis. Additionally, quantitative real‐time polymerase chain reaction (qRT‐PCR) was used to assess the level of *IL‐17RC* mRNA.

Results: The results revealed that carriers of the GA and AA genotypes of rs76999397 had a significantly increased risk of ALL (GA: odds ratios [OR] = 63.78, 95% confidence interval [CI] = 25.51–159.43, *p* < 0.0001; AA: OR = 18.22, 95% CI = 1.50–221.37, *p* < 0.0001). Additionally, we identified that an increased risk of ALL was associated with two haplotypes in *IL‐17RC*, C‐A and T‐A (in the order of rs708567 and rs76999397) (OR = 46.73, 95% CI = 17.30–126.28; OR = 49.42, 95% CI = 6.95–351.45, respectively).

Conclusions: The results suggested that the GA and AA genotypes of rs76999397 were significantly associated with an increased risk of ALL, whereas rs708567 did not show a significant association. Furthermore, the CA and TA haplotypes (rs708567/rs76999397) were found to be associated with increased susceptibility to ALL.

## 1 Introduction

The IL‐17 family comprises six cytokines: IL‐17A, IL‐17B, IL‐17C, IL‐17D, IL‐17E (IL‐25) and IL‐17F, interacting with five distinct receptors (IL‐17RA–IL‐17RE). These interactions are critical for the immune system's functioning (Sabry et al. 2020). Interleukin 17, a pro‐inflammatory cytokine produced by a specific T helper cell subset known as Th17, plays a pivotal role in the progression and pathogenesis of inflammatory diseases (Rodrigues‐Diez et al. 2021). Various cell types, including macrophages, dendritic cells, epithelial cells, fibroblasts and T lymphocytes, express IL‐17 receptors on their surfaces, featuring a common SEFIR domain internally and a fibronectin III‐like region externally (Ge et al. 2020).

Despite significant sequence variations, all IL‐17 receptors are single‐pass, Type I transmembrane glycoproteins. Notably, the extracellular domain of *IL‐17RC* is 55% longer than that of *IL‐17RA*, whereas its cytoplasmic segment is 44% shorter, sharing only 22% sequence identity. *IL‐17RC* is also distinguished by its multiple splice forms, unlike IL‐17RA (Goepfert et al. 2020). IL‐17 signalling requires the heterodimeric combination of A and C receptors, with the C subunit critically modulating responses (Ho and Gaffen 2010).

The IL‐17R complex, a crucial component of the IL‐17/IL‐17R signalling axis, facilitates the signal transduction necessary to produce pro‐inflammatory cytokines such as tumour necrosis factor α (TNF‐α), interleukin‐6 (IL‐6) and interleukin‐1β (IL‐1β) (Jiang et al. 2022). This complex not only regulates inflammation and disease development but also activates downstream responses upon cytokine‐receptor interaction (Korppi et al. 2020).

Emerging evidence suggests that dysfunction within the IL‐17RC component and the broader IL‐17/IL‐17R signalling axis is linked to various human diseases (Zhou et al. 2012). The rs708567 mutation, a common missense alteration (S111L) in the IL‐17RC gene, has been associated with several diseases, including systemic lupus erythematosus, rheumatoid arthritis, adolescent idiopathic scoliosis (AIS) and ossification of the thoracic posterior longitudinal ligament (Hristova et al. 2021). Genetic variations in the IL‐17 signalling pathway may undermine immune defences by impacting IL‐17 production and function. Such single nucleotide polymorphisms (SNPs) can alter gene expression and protein function, potentially leading to uncontrolled cell growth and the development of cancer (Alghamdi et al. 2022).

Acute lymphoblastic leukaemia (ALL) is a malignant disorder characterized by the unchecked proliferation of immature lymphoid cells within the bone marrow and peripheral blood. Primarily affecting children, yet not sparing adults, ALL is known for its rapid progression and challenging prognosis. The aetiology of ALL involves a complex interplay between genetic predispositions and environmental influences. A deeper understanding of the molecular foundations of ALL is crucial for the development of targeted therapeutic interventions and the enhancement of prognostic accuracy. Investigating the genetic and molecular irregularities that precipitate the onset and advancement of ALL, such as alterations in the *IL‐17RC* gene and its influence on cytokine signalling pathways, holds the promise of advancing personalized medicine. This refined understanding could improve therapeutic outcomes by facilitating more precise disease management and predicting individual responses to specific treatments.

## 2 Materials and Methods

The study cohort included 95 Saudi patients diagnosed with ALL (84 males and 11 females; age range: 1–41 years; mean age: 16.1 ± 11.7 years). The control group consisted of 95 healthy individuals (65 males and 30 females; age range: 1–81 years; mean age: 43.7 ± 22.1 years). The study received approval from the Ethics Committee of King Saud University, and all participants provided written informed consent (Ref. No. 20/0795/IRB).

Blood samples were collected from all participants using EDTA vacutainer tubes. Genomic DNA was isolated from 3 mL of whole blood from both ALL patients and healthy participants using the Qiagen Blood Mini Kit (Qiagen, Germany). During DNA extraction, the instructions provided with the kit were meticulously followed. The purity and quantity of the isolated DNA were assessed using a NanoDrop spectrophotometer (Thermo Scientific, Wilmington, DE, USA) at 260/280 nm wavelengths. The purity of the total DNA ranged from 1.7 to 2.0, and it was subsequently stored at −20°C.

### 2.1 Genotyping

For the *IL‐17RC* gene, we selected SNPs on the basis of the following criteria: minor allele frequency (MAF) ≥0.05 in the Saudi population; Hardy–Weinberg equilibrium (HWE) with a *p* value cut‐off >0.05; and SNPs located in functional regions (Table 1). The genotypes of SNPs at rs76999397 G/A and rs708567 C/T in *IL‐17RC* were determined using the TaqMan allelic discrimination method on a 96‐well ABI 7900HT Real‐Time PCR System (Applied Biosystems, Foster City, CA, USA) according to the manufacturer's instructions. All PCR reactions were carried out in a total volume of 10 µL, comprising 0.26 µL of 2× SNP genotyping assay, 5 µL of 2× Power Taq MasterMix, 2.24 µL of nuclease‐free water and 2 µL of DNA template (100 ng/µL). Amplification was performed under the following conditions: 50°C for 2 min, 95°C for 10 min, followed by 40 cycles of 95°C for 15 s and 60°C for 1 min. For confirmation, approximately 5% of samples were randomly selected for repeat genotyping.

**TABLE 1:** Characteristics of selected polymorphisms involved in the *IL‐17RC* rs708567 and rs76999397.

					MAF in human populations (1000 genomes study)				
Gene	SNP ID	Chromosome position	Nucleotide change	Region	Global	African	European	South Asian	East Asian
*IL‐17RC*	rs708567	chr3:9918386	C>T	Promoter	0.50	0.47	0.54	0.34	0.05
	rs76999397	chr3:9933125	G>A	Promoter	0.027	0.012	0.028	0.00	0.009

Abbreviations: MAF, minor allele frequency; SNP, single nucleotide polymorphisms.

### 2.2 Quantitative Real‐Time Polymerase Chain Reaction (qRT‐PCR) Analysis

Total RNA was extracted from fresh blood samples using the PureLink RNA Mini Kit (Invitrogen, Carlsbad, CA, USA) following the manufacturer's guidelines. The samples were stored at −80°C. RNA was converted to complementary DNA (cDNA) using the PrimeScript RT reagent kit (Takara Bio Inc., Dalian, China). The reverse transcription reaction was conducted at 37°C for 15 min, followed by 85°C for 5 s. qRT‐PCR was conducted using an ABI Prism 7500 Real‐Time PCR System (Applied Biosystems, Foster City, CA, USA) in accordance with the manufacturer's protocols (Schmittgen and Livak 2008). The reaction volume was 10 µL, comprising 5 µL of SYBR Green Real‐time PCR Master Mix, 3.2 µL of DEPC‐treated H_2_O, 1 µL of cDNA and 0.8 µL each of forward and reverse primers. PCR primers for *IL‐17RC* were designed using Primer Express 3.0 software (Applied Biosystems), with glyceraldehyde 3‐phosphate dehydrogenase (GAPDH) mRNA (GenBank accession number NM‐002046.7) serving as an internal control. The primers used were IL‐17RC: 5′‐AGTAGGGTAGGCCTGGAAGG‐3′ (forward) and 5′‐CACTGGGAAGAGCCTGAAGA‐3′ (reverse) and GAPDH: 5′ TCTCCTCTGACTTCAACAGCGAC‐3′ (forward) and 5′‐CCCTGTTGCTGTAGCCAAATTC‐3′ (reverse). Relative quantification of the data was performed using the comparative 2^−ΔΔCt^ method and normalized to the housekeeping gene GAPDH.

### 2.3 Statistical Analysis

The chi‐square test was used to check for adherence to the HWE and to compare the *IL‐17RC* genotype distribution and allele frequency between ALL patients and healthy controls. Odds ratios (OR) with 95% confidence intervals (CIs) were calculated. Logistic regression models were applied to evaluate potential associations between *IL‐17RC* genotypes using the web‐based SNPStats software programme (Solé et al. 2006). Co‐dominant, dominant and recessive models were utilized to investigate the association between the rs76999397 G/A and rs708567 C>T polymorphisms and the development of ALL. Statistical significance was set at a *p* value of <0.05.

## 3 Results

The frequency of different polymorphic genotypes and alleles for the studied SNPs (*IL‐17RC* rs76999397 G/A and rs708567 C>T) is illustrated in Table 2. All patients and controls were tested for rs76999397 G/A and rs708567 C/T polymorphisms. The genotype distributions of the two SNPs were consistent with the HWE test in control subjects (*p* > 0.05).

**TABLE 2:** Genotype distributions and allele frequencies of *IL‐17RC* (rs708567 and rs76999397) in cases and controls.

Locus	Model	Genotype	Control (%) *N* = 95	Case (%) *N* = 95	OR (95% CI)	*p* value	AIC	BIC
rs708567 C>T	Alleles	C	168 (0.88)	163 (0.86)	1.00			
		T	22 (0.12)	27 (0.14)	1.26 (0.69–2.31)	0.44		
	Co‐dominant	C/C	75 (79)	69 (72.6)	1.00	0.42	267.7	277.4
		C/T	18 (18.9)	25 (26.3)	1.51 (0.76–3.01)			
		T/T	2 (2.1)	1 (1.1)	0.54 (0.05–6.13)			
	Dominant	C/C	75 (79)	69 (72.6)	1.00	0.31	266.4	272.9
		C/T‐T/T	20 (21.1)	26 (27.4)	1.41 (0.72–2.76)			
	Recessive	C/C‐C/T	93 (97.9)	94 (99)	1.00	0.56	267.1	273.5
		T/T	2 (2.1)	1 (1.1)	0.49 (0.04–5.55)			
	Over‐dominant	C/C‐T/T	77 (81)	70 (73.7)	1.00	0.22	265.9	272.4
		C/T	18 (18.9)	25 (26.3)	1.53 (0.77–3.04)			
	Log‐additive	—	—	—	1.27 (0.69–2.32)	0.44	266.8	273.3
rs76999397 G>A	Alleles	G	176 (0.93)	102 (0.54)	1.00			
		A	14 (0.07)	88 (0.46)	**10.85 (5.86–20.05)**	**<0.0001**		
	Co‐dominant	G/G	82 (86.3)	9 (9.5)	1.00		140.9	150.6
		G/A	12 (12.6)	84 (88.4)	**63.78 (25.51–159.43)**	**<0.0001**		
		A/A	1 (1.1)	2 (2.1)	18.22 (1.50–221.37)			
	Dominant	G/G	82 (86.3)	9 (9.5)	1.00	**<0.0001**	139.7	146.2
		G/A‐A/A	13 (13.7)	86 (90.5)	**60.27 (24.45–148.56)**			
	Recessive	G/G‐G/A	94 (99)	93 (97.9)	1.00	0.56	267.1	273.5
		A/A	1 (1.1)	2 (2.1)	2.02 (0.18–22.68)			
	Over‐dominant	G/G‐A/A	83 (87.4)	11 (11.6)	1.00	**<0.0001**	144.2	150.7
		G/A	12 (12.6)	84 (88.4)	**52.82 (22.07–126.40)**			
	Log‐additive	—	—	—	**47.59 (20.01–113.15)**	**<0.0001**	146.8	153.3

*Note*: ALL acute lymphoblastic leukaemia, OR odds ratio, 95% CI 95% confidence interval, *p* < 0.05 was considered significant and are depicted in bold.

The genotype distributions for the *IL‐17RC* rs76999397 G/A polymorphism were significantly different between ALL patients and healthy controls. According to logistic regression analysis, the results revealed that carriers of GA and AA genotypes had a significantly increased risk with ALL populations (GA: OR = 63.78, 95% CI = 25.51–159.43, *p* < 0.0001; AA: OR = 18.22, 95% CI = 1.50–221.37, *p* < 0.0001). Thus, the GA mutant genotype may be considered a genetic risk factor in ALL. Similarly, in the dominant and over‐dominant model, the *IL‐17RC* rs76999397 G/A polymorphism was linked to an increased risk of ALL (GG vs. AG + AA: OR = 60.27, 95% CI = 24.45–148.56, *p* < 0.0001; GG + AA vs. AG: OR = 52.82, 95% CI = 22.07–126.40, *p* < 0.0001). Results also showed that the A‐allele mutant was more prevalent in ALL patients than in healthy controls, suggesting a risk effect of this allele (OR = 10.85, 95% CI = 5.86–20.05, *p* < 0.0001) in patients with ALL. The frequencies of the CC, CT and TT genotypes among patients were 72.6%, 26.3% and 1.1%, respectively, and in controls were 79%, 18.9% and 2.1%, respectively, for *IL‐* *17RC* rs708567 C>T. The comparison of these frequencies with those in the control group did not show a statistical difference (*p* > 0.05). Additionally, patients had a higher frequency of the mutant T‐allele than controls (0.14 vs. 0.12), but this increase was not statistically significant (*p* > 0.05).

### 3.1 Haplotype Analyses of *IL‐17RC* Gene Polymorphisms and ALL Risk

To account for two SNPs in the same gene, we performed a haplotype analysis to explore their combined genetic effects. As shown in Table 3, we found that haplotypes C‐A and T‐A (in the order of **rs708567** and **rs76999397**) were significantly associated with an increased risk of ALL (OR = 46.73, 95% CI = 17.30–126.28, *p* < 0.0001 and OR = 49.42, 95% CI = 6.95–351.45, *p* = 0.0001, respectively), compared with the most frequent haplotype, C‐G, which served as the reference haplotype.

**TABLE 3:** Combinations of genotyped SNPs in *IL‐17RC* (rs708567 and rs76999397) that show significant differences between acute lymphoblastic leukaemia (ALL) patients and controls, as determined by haplotype analysis.

rs708567	rs76999397	Control	Case	OR (95% CI)	*p* value
C	G	0.8241	0.4888	Ref	
C	A	0.2184	0.3691	**46.73 (17.30–126.28)**	**<0.0001**
T	G	0.0789	0.048	0.93 (0.21–4.13)	0.92
T	A	0.0501	0.0941	**49.42 (6.95–351.45)**	**0.0001**

*Note*: Data are shown as a number (percentage). OR: odds ratio (95% CI) (95% confidence interval).

To account for two SNPs in the same gene, we performed a haplotype analysis to explore their combined genetic effects. As shown in Table 3, we found that haplotypes C‐A and T‐A (in the order of rs708567 and rs76999397) were significantly associated with an increased risk of ALL (OR = 46.73, 95% CI = 17.30–126.28, *p* < 0.0001; and OR = 49.42, 95% CI = 6.95–351.45, *p* = 0.0001, respectively), compared with the most frequent haplotype, C‐G, which served as the reference.

### 3.2 Relative mRNA Expression of *IL‐17RC*

**FIGURE 1:**
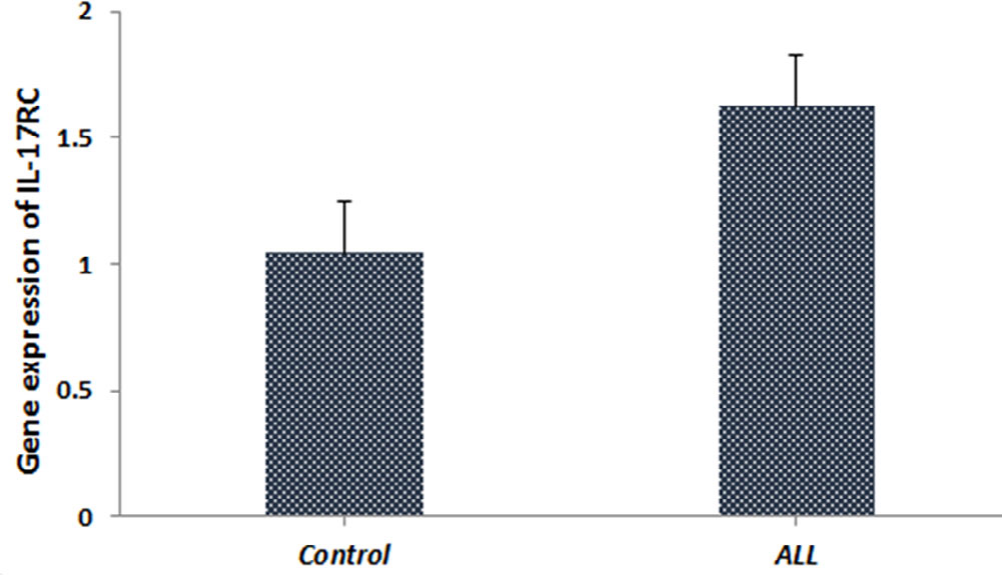
The relative expression of the *IL‐17RC* gene in ALL patients and healthy control using 2 ▵▵Ct method and normalized to the average of the GAPDH housekeeping gene.

The expression of the *IL‐17RC* gene in patients was assessed using qRT‐PCR. Analysis of the relative mRNA expression levels in ALL patients and healthy control subjects indicates higher levels in the control group than in the patients. However, the difference between the two groups is not statistically significant (*p* > 0.05) (Figure 1).

## 4 Discussion

ALL is the most common malignancy diagnosed in children and remains a significant health concern due to its increasing incidence in recent years (Malard and Mohty 2020). It is characterized by the uncontrolled proliferation of lymphoid progenitor cells in the bone marrow, blood, and extramedullary sites (Inaba et al. 2013). Despite substantial advancements in diagnostic techniques and treatment methods for ALL, its underlying aetiologies remain unclear (Zou et al. 2017).

The *IL‐17* receptor complex, primarily comprising *IL‐17RA* and *IL‐17RC*, is essential for IL‐17's biological activity and activates the NF‐κB and MAPK signalling pathways (Dhaouadi et al. 2018; Toy et al. 2006). Current data suggest that both IL‐17RC dysfunction and the IL‐17 cytokine/IL‐17R signalling axis are implicated in a variety of human diseases (Zhou et al. 2012). Due to its central role in inflammation regulation, dysfunctions in the IL‐17 cytokine/IL‐17R signalling axis can promote the production of several pro‐inflammatory cytokines, such as TNF‐α, IL‐1β and IL‐6 (Dhaouadi et al. 2018).

The IL‐17 family activates downstream responses by binding to cell surface receptors; for example, the binding of *IL‐17* is dependent on *IL‐17RC* (Song and Qian 2013). SNP rs708567 is a common missense mutation (Ser 111 Leu) located on chromosome 3p25.3 within an exon of the *IL‐17RC* gene, which codes for a crucial component of the IL‐17R complex. This complex mediates signal transduction for the *IL‐17* cytokine family, which triggers the production of cytokines that promote inflammation, such as *TNF‐α*, *IL‐6* and *IL‐1β* (Zhou et al. 2012).

Several studies have focused on *IL‐17RC* and reported its association with various diseases. Dormans et al. (2010) discovered that SNP rs708567 in the *IL‐17RC* gene is associated with increased susceptibility to AIS in a European population, suggesting that *IL‐17RC* may be an AIS susceptibility gene.

In this study, we investigated two SNPs of the *IL‐17RC* polymorphism (rs708567 C/T and rs76999397 G>A) in Saudi ALL patients and controls. To our knowledge, no previous study has examined the *IL‐17RC* SNP polymorphism in Saudi ALL patients. Our study investigated the frequency and potential risk modification of the *IL‐17RC* (rs708567 C/T) polymorphism genotype and allele on ALL susceptibility in the Saudi population. Our data did not reveal statistically significant associations of *IL‐17RC* (rs708567) in ALL patients and healthy control groups (*p* > 0.05).

These findings indicate that the rs708567 (C/T) polymorphism did not appear to impact susceptibility to ALL in the Saudi population. A similar lack of association was reported by Dhaouadi et al. (2018) in a study of Tunisian patients with rheumatoid arthritis, where no significant differences in IL‐17RC rs708567 genotypes and alleles were found between cases and controls.

Similarly, Takahashi et al. (2011) in Japan did not discover a link between this SNP and AIS in Japanese females. Moreover, the study by Sharma et al. (2011) which included 419 American AIS patients and their families, did not discover a relationship between the IL‐17RC gene polymorphism and susceptibility to AIS.

Conversely, Kariuki et al. (2013) discovered that rs708567 CC homozygotes had a lower risk of acute seizures in malaria (*p* = 0.007, OR = 0.78) compared to CT and TT genotypes. In a Chinese Han population, Zhou et al. (2012) found a strong link between the *IL‐17RC* gene polymorphism and AIS susceptibility and curve severity. The small sample sizes, variation in the control populations and racial and ethnic differences may contribute to the disparities observed in these studies’ assessments of this SNP. Regarding the *IL‐17RC*‐rs76999397 G>A gene, our findings revealed that in the analysed population, the GA and AA genotypes of the investigated *IL‐17RC* (rs76999397) polymorphism increased the risk of ALL compared to the GG genotype. Thus, both the GA heterozygous and AA homozygous genotypes may be considered genetic risk factors in ALL. Similarly, the rs76999397 polymorphism was associated with an increased risk of ALL in both the dominant and over‐dominant models. Additionally, the A‐allele was more prevalent in ALL patients than in controls, suggesting this allele is a risk factor for patients in Saudi Arabia with ALL.

Our results were in agreement with the only study that found the A allele frequency of rs76999397 was higher in T‐OPLL patients (thoracic ossification of the posterior longitudinal ligament) than in controls (OR, 4.6; 95% CI, 0.99–21.87; *p* = 0.03), indicating that the A allele might be a risk factor for genetic susceptibility to T‐OPLL and that the GA genotype increased the probability of T‐OPLL susceptibility (Wang et al. 2018).

In the current study, the results revealed a decrease in mRNA levels in ALL patients compared to the healthy control group, but these results were not statistically significant. The observed variations, however, were not statistically significant. Although there is a trend, this lack of statistical significance may indicate that the differences in mRNA levels could be caused by biological variability, sample size or the complexity of gene regulation in ALL.

Finally, the results of our haplotype analysis demonstrated that the haplotypes CA and TA (in the order of rs708567 and rs76999397) were more frequent in patients with ALL. These haplotypes may serve as indicators of increased genetic risk for ALL. However, our study has some limitations. The sample size of ALL patients was modest, as this was a pilot study. Larger cohorts are required to confirm these genotype‐phenotype associations and to better understand the roles of rs76999397 (G/A) and rs708567 (C/T) polymorphisms. Further functional studies are also needed to elucidate the molecular mechanisms by which these SNPs might influence immune regulation and protein function. Additionally, future multivariate analyses incorporating clinical variables may provide a more comprehensive understanding.

## 5 Conclusions

To the best of our knowledge, this is the first association study investigating ALL susceptibility genes in Saudi patients. The CA and TA haplotypes (in the order of rs708567 and rs76999397) in the *IL‐17RC* gene were significantly associated with an increased risk of ALL. Our findings suggest that the GA and AA genotypes of the rs76999397 SNP, but not rs708567, may contribute to ALL susceptibility in this population. To validate these findings, further large‐scale genetic studies involving diverse populations, along with functional analyses, are needed.

## Conflicts of Interest

The authors declare no conflicts of interest.

## Data Availability

The data that support the findings of this study are available on request from the corresponding author. The data are not publicly available due to privacy or ethical restrictions.
